# A Robust Lane Detection Model Using Vertical Spatial Features and Contextual Driving Information

**DOI:** 10.3390/s21030708

**Published:** 2021-01-21

**Authors:** Wenbo Liu, Fei Yan, Jiyong Zhang, Tao Deng

**Affiliations:** School of Information Science and Technology, Southwest Jiaotong University, Chengdu 611756, China; liuwenbo@my.swjtu.edu.cn (W.L.); fyan@swjtu.edu.cn (F.Y.); zhangjiyong@my.swjtu.edu.cn (J.Z.)

**Keywords:** lane detection, vertical spatial features, contextual information, complex driving scenes

## Abstract

The quality of detected lane lines has a great influence on the driving decisions of unmanned vehicles. However, during the process of unmanned vehicle driving, the changes in the driving scene cause much trouble for lane detection algorithms. The unclear and occluded lane lines cannot be clearly detected by most existing lane detection models in many complex driving scenes, such as crowded scene, poor light condition, etc. In view of this, we propose a robust lane detection model using vertical spatial features and contextual driving information in complex driving scenes. The more effective use of contextual information and vertical spatial features enables the proposed model more robust detect unclear and occluded lane lines by two designed blocks: feature merging block and information exchange block. The feature merging block can provide increased contextual information to pass to the subsequent network, which enables the network to learn more feature details to help detect unclear lane lines. The information exchange block is a novel block that combines the advantages of spatial convolution and dilated convolution to enhance the process of information transfer between pixels. The addition of spatial information allows the network to better detect occluded lane lines. Experimental results show that our proposed model can detect lane lines more robustly and precisely than state-of-the-art models in a variety of complex driving scenarios.

## 1. Introduction

Due to the development of deep learning, we can use convolutional neural networks to process images and help us solve some of the problems encountered in the field of unmanned driving, such as the lateral control of the car [1], prediction of driver focus [2], and understanding of traffic driving scenes. Lane detection [3] is an important part of how unmanned vehicles understand traffic driving scenes. Lane detection can provide increased information about the driving environment to help vehicles make high-quality driving decisions.

**Figure 1:**
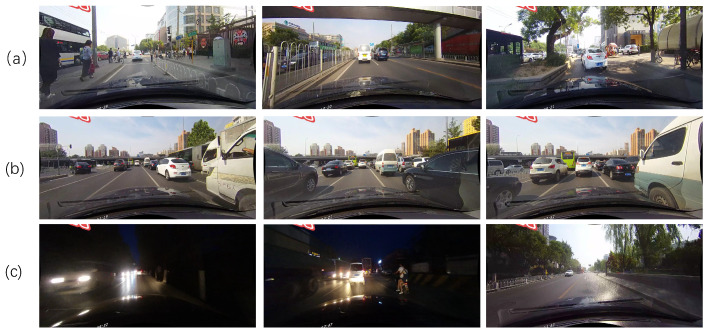
Examples of complex traffic driving scenarios. (**a**) Lane lines with different appearances. (**b**) Dynamic driving scenes. (**c**) Complex surrounding environment.

There are many difficulties encountered when performing lane detection in complex traffic driving scenes. Some examples of complex traffic driving scenarios are shown in Figure 1. First, the appearances of lane lines in different driving scenarios can be different, such as fences and steps (Figure 1a). When facing these scenarios, many algorithms cannot predict lanes accurately. Second, the surrounding environment of the driving vehicle changes as the vehicle travels. Figure 1b shows that the lanes on both sides of the vehicle are gradually blocked by other vehicles in the process of the vehicle moving forward. When the vehicle is in its daily driving pattern, it encounters many similar phenomena that bring difficulties to lane detection. Finally, lane detection is also affected by the current environment (Figure 1c). In many harsh environments, it is difficult for vehicles to clearly find lanes; such environments include poor light and dazzling light. Therefore, lane detection is a very challenging task in the field of unmanned driving.

Lane detection algorithms can be divided into two categories: one is based on image features that are extracted and then fitted [4], and the other is based on the deep learning method. In the former category, most algorithms need to extract image features, such as color features [5] and edge features [6]. Next, some other algorithms, such as the random sample consensus (RANSAC) [7] and Hough transform [8] methods, are commonly used to help with lane detection. However, this kind of method is often dependent on the selection of specific features in specific scenes to obtain good results, and this is difficult to achieve in complex driving scenarios. Cars that can perform autonomous driving have high requirements in terms of understanding driving scenarios, especially for lane detection in various scenarios. Lane detection based on deep learning [9] has great advantages in this respect. The deep convolutional neural network can be trained to perform semantic segmentation on scene pictures to understand the scene [10,11]. Therefore, lane detection based on deep learning exhibits a strong advantage with regard to autonomous driving.

**Figure 2:**
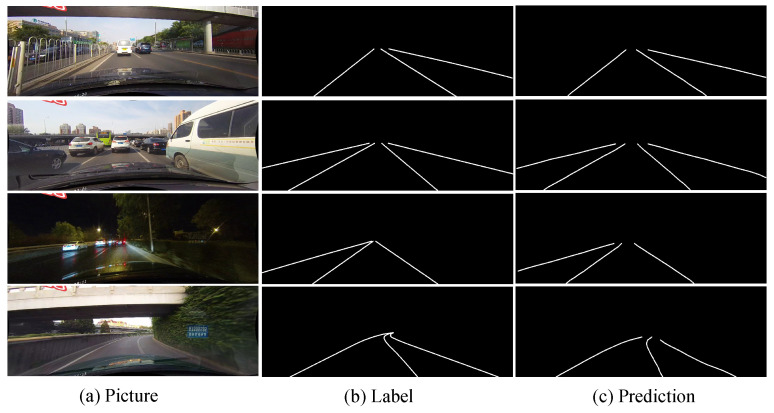
The lane detection results of our network in a variety of complex traffic scenarios on CULane dataset.

In this paper, we propose a deep convolutional neural network to detect lane lines in a variety of complex traffic scenarios. We enhance the accuracy of lane detection by the network for complex scenes by increasing the amount of contextual information and enhancing the transmission of information between pixels. In general, the feature map may lose contextual information after the convolution operation in the non-bottleneck part of the network. Therefore, we design a feature merging block that includes a skip layer and factorized convolutions [12] to allow the subsequent network to receive increased contextual information. Spatial convolutions [13] can strengthen the information transmission between adjacent layers in the feature map, and dilated convolutions can increase the size of the receptive field of the network. Therefore, we combine their advantages to design an information exchange block for enhancing the effective transmission of information between pixels. The final lane detection results of our proposed network are shown in Figure 2c. Faced with the difficulties of lane detection we mentioned before, our network can still effectively predict unclear or blocked lanes. This work is based on our previous work accepted by a conference proceeding [14], which provides more performance analysis and experimental demonstrations.

In summary, our main work is as follows.

1. We propose a robust lane detection model using vertical spatial features and contextual driving information that works well for unclear or occluded lanes in complex driving scenes, such as crowded scene, poor light condition, etc.
2. The information exchange block and feature merging block are designed to make the proposed model use the contextual information effectively and only use the vertical spatial features combined with lane line distribution features to robustly detect unclear and occluded lane lines.
3. We present many comparisons with other state-of-the-art lane detection models on the CuLane and TuSimple lane detection datasets. The experimental results show that our proposed model can detect the lane lines more robustly and precisely than others in the complex driving scenes.

## 2. Related Work

Early lane detection works were mainly used to assist the driving process of the vehicle. At that time, lane detection did not require a deep understanding of traffic scenes and only required the detection of ego lanes to meet the demands of applications. Beyeler et al. [15] proposed a real-time approach for ego-lane detection. Cooperation between the RANSAC method and a ridge operator can improve the efficiency of lane detection. Many algorithms based on simple scenes propose using image features to detect lanes and obtain good results. Kamble et al. [16] proposed using the Canny edge detection algorithm to detect the edges of roads and dividers while simultaneously using Huff transform technology to refine the edges. Then, some people began to detect lanes in some complex scenes. Wennan et al. [17] proposed a real-time algorithm based on a hyperbola-pair model, and their experimental results show that the algorithm performs robustly when there are arrow signs and colorful lane markings on the road. Yim et al. [18] proposed a three-feature-based automatic lane detection algorithm (TFALDA) that uses the optimal weighted combination of the starting position, direction, and gray-level intensity features comprising a lane vector to predict lane lines very efficiently. Wang et al. [19] proposed a lane detection model based on the B-snake model, which does not require any camera parameters, can describe a wide range of lane structures, and is robust to some complex scenes.

Xing et al. [20] compared the representative studies on lane detection systems and discussed the limitations of the lane detection systems available at that time. Vehicles’ understanding of traffic scenes is becoming increasingly important, especially in the case of autonomous vehicles. With the rapid development of deep learning, an increasing number of people have proposed methods based on deep convolutional networks to solve the problem of lane detection. Pan et al. [13] proposed that spatial CNNs can more efficiently learn the spatial relationships of feature maps and the smooth, continuous priors of lane lines in traffic scenarios than previously developed networks. Spatial CNNs use residuals for the information transfer process, and their experimental results can prove that the message transfers of spatial CNNs are better than those based on long short-term memory (LSTM). Hou et al. [21] proposed a self-attention distillation (SAD) network model, which can allow the network to learn from itself without additional supervision or labels. The SAD module is added to the training process, and not only can it modify the attention map of the shallow block and extract rich contextual information, but also the enhanced features learned by the shallow block affect the deep block, thereby improving the accuracy of the final result. Ghafoorian et al. [22] proposed a network framework using generative adversarial networks (GAN) to identify lane lines. By sending the ground truth to the discriminative model and sending the original image to the generative model, the trained generative model can generate a lane line prediction map. Wang et al. [23] and others proposed a LaneNet network framework; the decoder in LaneNet is divided into two branches: the embedding branch and segmentation branch. Finally, the results of the two branches can be combined to obtain the result of instance segmentation. Neven et al. [24] designed an HNet, which can cope with changes in the number and shape of lane lines, to learn the transformation matrix of the aerial view transformation under LaneNet’s network structure. Ko et al. [25] proposed a multilane detection method based on the deep learning method, and the architecture of the method can reduce some error points generated by the network, thereby improving network performance. Liang et al. propose a LineNet for lane detection, which mainly contains the Line Prediction (LP) layer and the Zoom module [26]. The LineNet can understand the driving scenarios from multiple dimensions, such as position, direction, distance, type, etc.

Furthermore, some people use the advantages of recurrent neural networks to process continuous time information for lane detection. Li et al. [27] used a recurrent neural network to process sequential visual cues to detect lane boundaries, and used a multi-task convolutional neural network to predict the geometric attributes of the target. Yang et al. [28] used long short-term memory and recursive neural network to study spatial information can improve the performance of the model in complex scenarios. Zou et al. [29] analyzed the structure of lane lines and proposed a network structure combining convolutional neural network and recurrent neural network that can use information in continuous driving scenes for lane detection. Zhang et al. [30] proposed to use double ConvGRUs to better combine the advantages of deep convolutional neural networks and time information for lane detection.

## 3. Network Architecture

**Figure 3:**
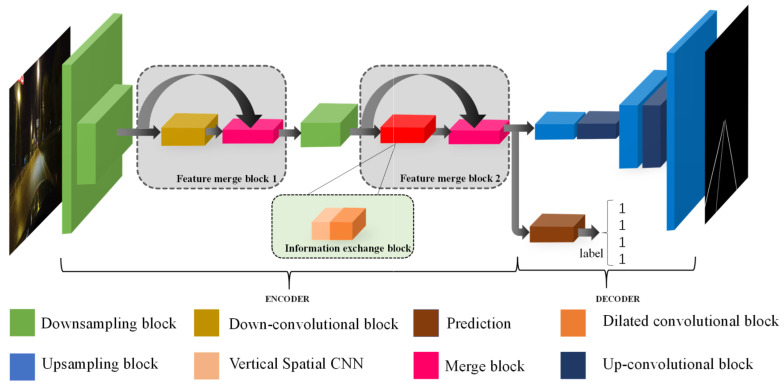
The architecture of the lane detection network.

In this section, we describe the proposed network architecture used to detect lanes in detail. The network architecture is shown in Figure 3. Inspired by the ERFNet [31], we propose an end-to-end network that performs pixel-level prediction to detect lanes. When a driving scene image is input into the network, the network can output the predicted position of the lane in the image. Our network consists of an encoder and two decoders, and the function of the two decoders is to predict the existence of lanes and the probability map of lanes. Therefore, we introduce the entire network framework from the points of view of these four parts of the network ((A) Encoder; (B) Prediction of the probability map; (C) Prediction of the existing lanes; and (D) Loss function).

### 3.1. Encoder

The shape of a lane is thin and long, and it accounts for a low proportion of the pixels in a traffic scene image. This requires that the quality of the feature map generated by the encoding network is high and that the feature map contains enough context information. In this section, we discuss several important blocks in the encoding network. The structure of the encoding network is shown in Table 1.

#### 3.1.1. Downsampling and Non-Bottleneck-1D Blocks

**Figure 4:**
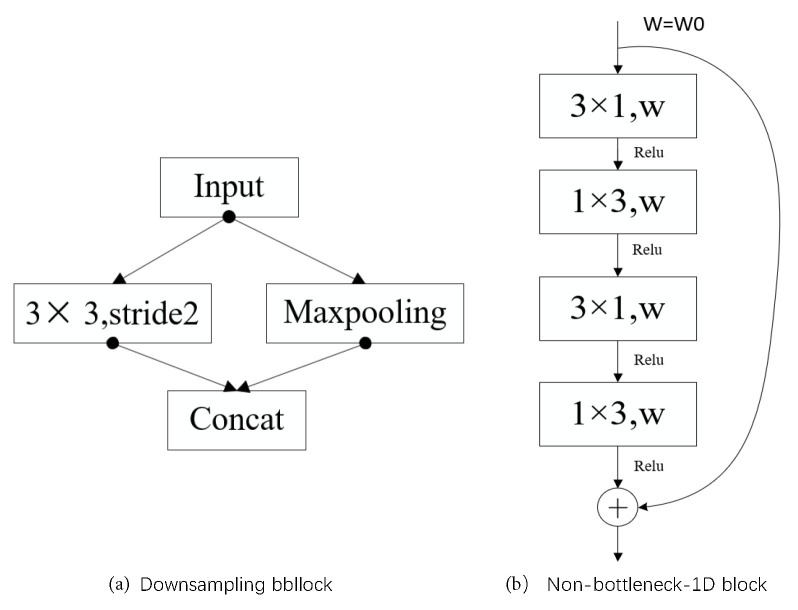
(**a**) The downsampling block. (**b**) The non-bottleneck-1D block.

The semantic information of the feature map output by the encoding network is critical to the quality of the lane detection network. The larger the receptive field of the feature map is, the richer the semantic information. Downsampling is very useful for increasing the receptive field of the feature map [32]. Increasing the width and depth of the network in parallel can enable the sharing of the computing pressure of the network from two dimensions to improve the effect of the network [33]. Therefore, the maxpooling layer and the convolutional layer are used in parallel in the downsampling block (layers 1, 2, and 5) [34] in Figure 4a. It can not only speed up the inference time of the network, but also transmit an increased amount of contextual information to the subsequent network.

The downsampling operation increases the depth of the network, and the features extracted from the network are more abundant and have more semantic information than other networks. Simply increasing the depth of the network may cause the problems of vanishing/exploding gradients, so it is necessary to add a deep residual learning framework to optimize the network [35]. Based on this idea, the Non-block-1D block in Figure 4b) is used multiple times in the network. Non-bottleneck-1D uses multiple nonlinear activation functions *ReLU* [36] and uses multiple convolutions with 1D filters instead of 2D filters [12] to reduce the number of parameters and make the network easy to train. The down-convolution block (layer 3) is composed of five non-bottleneck-1D blocks.

#### 3.1.2. Feature Merging Block

**Figure 5:**
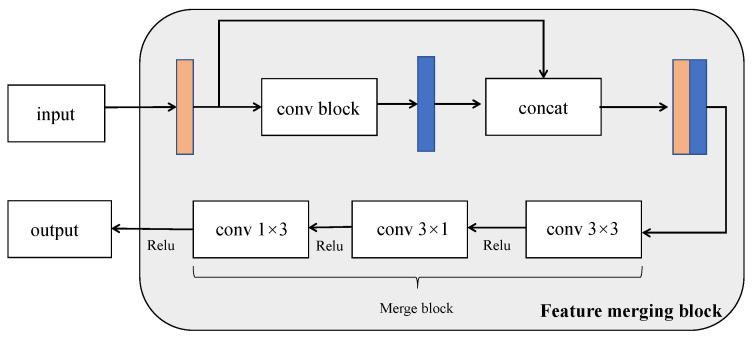
The architecture of feature merging block. The conv block of the feature merging block 1 (Figure 3) is the down-convolution block and the conv block of the feature merging block 2 (Figure 3) is the information exchange block.

When the lane is not clear or is occluded, contextual information is very important for lane detection. For example, if the lane is damaged, the model can only obtain the location of the lane through contextual information. With regard to the encoder–decoder network framework [37], contextual information is easily lost with the decrease in feature map resolution. Therefore, we design a feature merging block that can transmit more contextual information to the subsequent network together with downsampling. The feature merging block in the non-downsampling part of the encoding network is shown in Figure 5. Enough useful information can be transmitted to the following network so that the model can understand the information more efficient in the input image.

The input of the feature merging block is the feature maps output from the previous downsampling block and then output to the successive network, as shown in Figure 3. The conv block of the feature merging block is different for different positions of the overall block. Because the semantic information richness of the feature maps in different locations is different, the operation of extracting features should be different. The feature merging block transfers contextual information mainly in the non-downsampling part of the encoding network. As the model is supplemented with context information during the downsampling process, it is possible to continuously supplement the context information of the feature map during the entire encoding stage. If the two feature maps are added, the semantic information of each pixel can be increased, but for lane detection, which is a type of unbalanced semantic segmentation, this addition increases the error probability for pixels that are not lanes. The feature map processed by the conv block may cause the loss of local semantic information, and the concatenation operation can effectively supplement the contextual information.

Next, the feature map enters the merge block to achieve dimensionality reduction and feature merging. In the merge block (layers 4 and 8), the convolution is used to reduce the dimension of the block, and then multiple factorized convolutions are used for feature merging. We use factorized convolution because it can be used without changing the original input and output, and it can reduce the parameters and reduce the inference time required by the network. Finally, the encoded feature map can obtain more context information by the feature merge block.

#### 3.1.3. Information Exchange Block

Spatial convolution can promote the flow of information between adjacent layers of the feature map. The information of each layer is transmitted to the next layer by using the spatial convolution. Inspired by the principle of spatial convolution, we can not only focus on the relationship between the pixels in the receptive field, but also pay attention to the relationship between the spatial information of the pixels in the feature map over a large range. Lane lines mainly appear in the driving scene in a long continuous shape, so we consider adding vertical spatial features to the model by vertical spatial convolution. The vertical spatial feature can make our proposed model is more robust to detect occluded lane lines in complex driving scenes. Moreover, lane lines account for a low proportion of the pixels in a traffic scene image, we use dilated convolution to alleviate the problem of spatial feature information redundancy.

Dilated convolution can also increase the size of the receptive field of the feature map [32]. We can use dilated convolutions with different dilation rates to obtain spatial features with different scales for lane detection. In the process of using dilated convolution, it is necessary to pay attention to a hidden problem that some information in the feature map is not used. This is because the dilation rate is too large. As the convolution kernel moves, it cannot be calculated with every pixel of the feature map. Exchange pixel information before using dilated convolution by vertical spatial convolution, it can not only maintain the advantages of dilated convolution, but also prevent the loss of detailed information caused by the use of dilated convolution.

Vertical spatial convolution and dilated convolution can mutually improve performance. Therefore, we propose an information exchange block to make the model use the vertical spatial features better, and this block is composed of the vertical spatial convolution and the multiscale dilated convolution. This block is located after the third downsampling, and this greatly reduces the amount of computing resources used. As we mentioned earlier, the feature merge block allows an increased amount of context information to be passed to the subsequent network, and the information exchange block can extract the vertical spatial features to detect unclear and occluded lane lines with contextual information.

Finally, we discuss some details about the multiscale dilated convolution and the vertical spatial convolution. The use of dilated convolution is mainly based on the Non-bottleneck-1D block in the information exchange block. By combining the dilated convolutions of different dilation rates, the effect of dilated convolution is improved. We use the dilation rates of [1, 2, 1, 4] with the non-bottleneck-1D three times in the dilated convolutional block (layer 7). The dilation rates [a, b, c, d] indicate that four non-bottleneck-1D blocks are used, and the dilation rate of convolution in each block corresponds to [a, b, c, d]. Vertical spatial convolution (layer 6) is equivalent to proposing to train a 3-D kernel tensor K to transmit spatial information in the vertical direction and then pass it in the form of residuals layer by layer. It is verified by experiments that this convolution method can indeed effectively promote the flow of spatial information. Taking the downward spatial convolution as an example, the formula [13] can be expressed as

(1) $$\begin{array}{c} X_{i,j,k}^{\prime }=\left\{\begin{array}{c} X_{i,j,k},\;\;\;\;\;j=1\\ X_{i,j,k}+f\left(\sum_{m}\sum_{n}X_{m,j-1,k+n-1}^{\prime }\times K_{m,i,n}\right),\;\;j=2,3,\ldots ,H \end{array}\right. \end{array}$$

where $X_{i,j,k}$ is the element in the *i*-th channel, *j*-th row, and *k*-th column in the feature map; $X_{i,j,k}^{\prime }$ is obtained after $X_{i,j,k}$ is updated; and *f* is a nonlinear activation function as ReLU.

### 3.2. Prediction of the Probability Map

**Figure 6:**
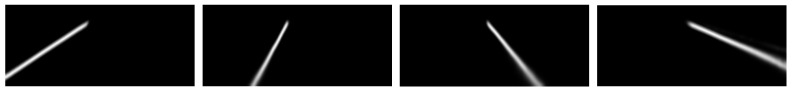
Predicted lane line probability pictures. We can see that different lane lines are predicted for different feature map channels, and some key points are selected and reconnected to obtain the final prediction map.

The task of this decoding branch is to enlarge the size of the feature map, fine tune the details [34], and finally generate the probability map. In the same single-channel probability map, it is difficult to determine which lane a specific pixel belongs to, so the network outputs different lanes to different channels. The resulting lane prediction map is shown in Figure 6. The probability map generated in this way can easily evaluate the quality of the generated lane.

In this branch, we use the decoding structure in ERFNet [31], which performs upsampling by using transposed convolution and a Non-bottleneck-1D block. This decoding branch mainly refers to the decoding structure of ERFNet. Its specific decoding structure is shown in Table 2.

### 3.3. Prediction of the Existing Lanes

Inspired by the work of Pan et al. [13], we use a branch to predict the probability of the existence of lanes in the picture. In this branch, the feature map channel is first reduced by convolution, and then a linear function is used multiple times to predict the position relationship with other lanes and the existence of lanes.

This branch has two functions: (1) The process of weighting the loss of the road branch according to the total loss helps the network predict the lane probability map. The position relationship with other lanes and the existence of lanes have certain impacts on lane detection. (2) We replace the continuous lane with a series of points at fixed intervals. According to this branch, we can accurately select some points from the lanes on the probability map to accurately generate the final prediction map. In this way, the final prediction map has few error points, and the fitting effect of the curve is improved.

### 3.4. Loss Function

First, we introduce the loss function of the decoding branch for predicting the existence of lane lines. We use binary cross-entropy loss, which can solve the problem of binary classification. The main formula is

(2) $$\begin{array}{c} loss_{exist}\left(x_{i},y_{i}\right)=-\frac{1}{N}\;\sum_{i=1}^{N}\left[y_{i}\log x_{i}+\left(1-y_{i}\right)\log\left(1-x_{i}\right)\right] \end{array}$$

where $x_{i}$ denotes the probability that the *i*-th element is predicted to be a positive sample, $y_{i}$ denotes the label of the *i*-th element, and *N* denotes the number of pixels.

The next step for the semantic segmentation of lane lines is to apply cross-entropy loss ($loss_{ce}$); then, the loss used by the lane detection decoding branch can be expressed as

(3) $$\begin{array}{c} loss=loss_{ce}\left(s,s^{\prime }\right)+0.1*loss_{exist} \end{array}$$

where $s^{\prime }$ denotes the predicted probability map generated by the network and s denotes the ground truth corresponding to the predicted probability map.

## 4. Experiments

In this section, we verify the effectiveness of the proposed model through a large number of experimental results on TuSimple lane detection challenge (https://github.com/TuSimple/tusimple-benchmark/tree/master/doc/lane_detection) and CULane lane detection datasets.

### 4.1. Dataset

#### 4.1.1. TuSimple Lane Detection Benchmark Dataset

The frames of this dataset were collected on the highway during the day. Its driving scene is relatively simple, aiming at the detection of multiple lanes. There are 6408 frames in total, including 3268 frames for the training sets, 358 frames for the verification sets, and 2782 frames for the test sets.

#### 4.1.2. CULane Dataset

This dataset is a collection of a total of 133,235 frames, divided into 88,880 frames for the training set, 9675 frames for the verification set, and 34,680 frames for the test set. Pan et al. [13] proposed that it was important that the lane detection algorithms can detect the lane lines from the image context, so they manually used tools to annotate the lane lines based on the context information with cubic splines. The corresponding lane detection data in the dataset provide one normal scene and 8 challenging categories: normal, crowded, night, no line, shadow, arrow, dazzle light, curve, and crossroad. The proportion of each scene in the test dataset is shown in Table 3. The driving scene used for this dataset is very complex, aiming at the detection of 4 lanes.

### 4.2. Evaluation Metric

#### 4.2.1. TuSimple Lane Detection Benchmark Dataset

The TuSimple database uses accuracy as its evaluation metric. The accuracy is calculated by

(4) $$\begin{array}{c} accuracy=\frac{\sum_{clip}C_{clip}}{\sum_{clip}S_{clip}} \end{array}$$

where $C_{clip}$ is the number of lane points predicted correctly and $S_{clip}$ is the total number of ground truths in each clip.

#### 4.2.2. CULane Dataset

The CuLane database takes the F1-measure as its evaluation metric. We consider a predicted lane line pixel width of 30 pixels as a successfully predicted lane line and calculate the intersection-over-union (IoU) between the predictions and their corresponding ground truths. Lanes with calculated IoU values greater than a certain threshold are considered to be true positives (TP). After obtaining the TP, it is easy to identify false positives (FP) and false negatives (FN) according to the pixel information of the prediction map and the ground truth. According to their formulas, the following two indicators can be obtained:

(5) $$\begin{array}{c} Precision=\frac{TP}{TP+FP} \end{array}$$

(6) $$\begin{array}{c} Recall=\frac{TP}{TP+FN} \end{array}$$

These two indicators require our comprehensive consideration, so the most common method is to use the F1-measure to weigh the relationship between the two indicators. In the end, we use the F1-measure as the final evaluation indicator. The formula is

(7) $$\begin{array}{c} F_{1}=\frac{2\times Precision\times Recall}{Precision+Recall} \end{array}$$

### 4.3. Ablation Study

In this section, we perform experiments on the CuLane dataset to prove the rationality of the proposed network. We train the network model with 200 epochs to verify the results. At the same time, the selected IoU refers to the threshold for filtering out the predicted lane lines; the higher the IoU threshold is, the higher the quality of the predicted lane line. F1(m) denotes F1-measure that IoU threshold is m. Generally, F1 (0.3) and F1 (0.5) are selected for experiments to facilitate comparison with other models using the same data set [13,21,22].

#### 4.3.1. Comparison with Similar Models

Our proposed network model is inspired by ERFNet [31], and vertical spatial convolution is used in the information exchange block. As shown in Table 4, we chose the following three similar models for comparison with our network: ERFNet, SCNN [13], and ERFNet with horizontal and vertical spatial convolution operation (ERFNet + SCNN).

The experimental results shown in Table 4 indicate that when the selected IoU threshold is 0.3, the F1 measurement value of our network is lower than that of SCNN, but when the threshold value is 0.5, our model detects the lane most accurately. This is because SCNN can predict that there are many points near the lane, but when the selected IoU threshold is increased, these points are removed because they are too far from the lane. The evaluation scores of ERFNet and “ERFNet + SCNN” in both cases are flower than our proposed model. This shows that the lane detection process of our proposed model is effective in complex traffic scenarios, and the information exchange block and feature merging block can indeed play active roles in lane detection.

#### 4.3.2. Different Dilated Convolution Rates

The designed information exchange block is composed of a vertical spatial convolution and a multiscale dilated convolution. The purpose of this experiment is to discuss the use of dilated convolution with different dilation rates in the designed information exchange block. As mentioned earlier, our use of dilated convolution is based on the non-bottleneck-1D block. Next, we carry out three groups of experiments with different dilation rate combinations in Table 5.

In this experiment, we compare the effects of different dilation rate combinations for the dilated convolution blocks on the results. As shown in Table 5, comparing the dilation rates [2, 4, 8, 16] and [1, 2, 1, 4], it can be found that if the dilation rate is too large, the F1-measure decreases. The proportion of pixels of lanes in the prediction map is very small, and a large dilation rate makes the dilated convolution obtain useless features. Comparing the dilation rates [1, 1, 1, 1] and [1, 2, 1, 4], it can be found that multiscale dilated convolution is indeed effective. Compared with regular convolution, multiscale dilated convolution can obtain more effective contextual information. The final results show that dilated convolution with an appropriate dilation rate [1, 2, 1, 4] combination can improve the performance of lane detection.

#### 4.3.3. Method Used for Spatial Convolution

The spatial convolution module is located in the top hidden layer, and the feature information it contacts is sufficient. In the information exchange block, we use spatial convolution to enhance the information exchange between layers in the feature map. As shown in Table 6, the following experiments test how we can better use spatial convolution to fit our model.

As we can see from Table 6, the experimental results show that the use of vertical spatial convolution can improve the lane detection effect. There are two reasons for this: (1) The dilated convolution block is more suitable for extracting the semantic information of pixels in the feature map than the vertical spatial convolution. (2) Lanes are distributed vertically in the various traffic scenarios. The horizontal information in the feature map is greatly affected by the traffic environment, while the vertical information based on the lane distribution is stable.

### 4.4. Qualitative and Quantitative Comparisons

In this section, we compare the proposed model with other lane detection models on the TuSimple and CULane lane detection datasetsfrom two points of view: a qualitative evaluation and a quantitative evaluation.

#### 4.4.1. Qualitative Evaluation

**Figure 7:**
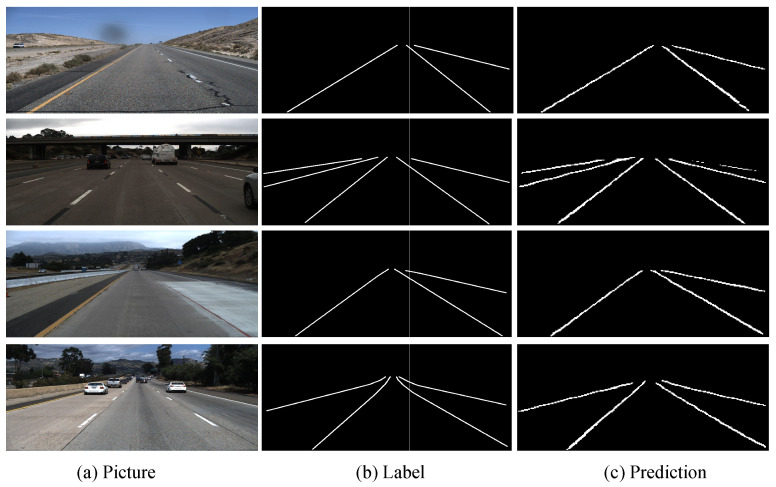
The results of the proposed network models on TuSimple.

**Figure 8:**
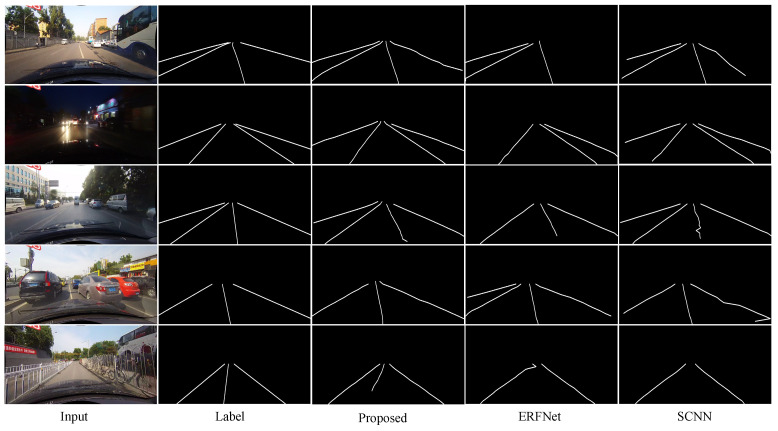
Comparison of the results with different network models on CULane dataset.

1. TuSimple lane detection benchmark dataset
  As shown in Figure 7, our model can accurately detect the lane lines on TuSimple dataset. As shown in the first row in Figure 7, even if the lane is damaged, it can be clearly detected by our model. This result indicates that the proposed model can use effective context information to robustly detect lane lines when the lane lines are unclear. The third row in Figure 7 shows our proposed model is not disturbed by the white line on the wall. Our proposed model still detects the lanes in the scene stably, this result also shows that our network has strong robustness to lane detection in complex driving scenarios. As shown in the last row in Figure 7, our model can accurately detect the lanes in simple driving scenarios.
2. CULane dataset
  Figure 8 shows the comparison of the lane detection results obtained by different models on CULane dataset. By comparing the output results of ERFNet and the basic SCNN, we can find that the quality of the line detected by ERFNet is better. However, due to its lack of understanding of the driving environment, it detects fewer or more lines than SCNN (the first four rows in Figure 8). Comparing these results with the experimental results of our proposed network, we can see that our model obtains a good combination of the advantages of the two. Some unclear (the second and last rows in Figure 8) and occluded (the first and forth rows in Figure 8) lanes also can be detected by our model precisely, but other models fail to these detections more or less. The use of vertical spatial features and contextual driving information has a more robust effect on the lane detection of our proposed model in complex driving scenes with occlusions and unclear lane lines. Our method has a good understanding of the traffic driving scenes, and the quality of the detected lines is higher than those of the other methods (the last row of Figure 8).

#### 4.4.2. Quantitative Evaluation

1. TuSimple lane detection benchmark dataset
  According to the evaluation scores shown in Table 7, we can see that our model can effectively perform lane detection in normal scenarios. However, the value of FP is the highest, indicating that our model is most likely to detect places that are not lanes in the labeled map as lanes. Like the second row in Figure 7, the rightmost lane does not appear in the labeled map, but our network can detect it. The proportion of challenging driving scenes on TuSimple dataset is small, but the proposed model is still robust to lane detection. Especially our proposed model performs better in some complex driving scenarios with unclear or occluded lane lines.
2. CULane dataset
  In Table 8, we compare the effects of different models on the CULane dataset and compare the lane detection effect for each scenario in the form of the F1-measure. It can be seen that our model is superior to other models in most driving scenarios. We can find that the effect of lane detection in normal scenes is significantly improved. The proposed model obtains the best lane detection results in the complex driving scenes of no line, shadow, arrow and dazzle light. Moreover, in the complex driving scenes of crowded and night, our model is very close to the optimal model. Our proposed model can achieve excellent performance in complex driving scenarios including unclear and occluded lane lines, which prove that the addition of vertical spatial features and effective context information can make the proposed model more robust to detection the lane lines. More contextual information obtained by the feature merge block and vertical spatial features extracted by the information exchange block can be fully utilized to make the proposed model with a strong ability to understand the complex driving environment. Furthermore, the total F1 (0.5) score of our proposed model is the highest in the Table 8. Recently, Liang et al. proposed the LineNet [26] can get the total F1-measure result of 73.1% that is a little higher than ours. However, they did not publish each scenario result and the source codes yet, so we cannot make more in-depth qualitative and quantitative comparisons with LineNet.

In short, the qualitative and quantitative experiments on TuSimple and CULane datasets show that our proposed model can robustly detect lanes in complex driving scenarios.

## 5. Discussion

According to the results in Table 8, the proposed model is not ideal for detecting curved lane lines and detecting lane lines on the crossroads. Therefore, we discuss the detection effect of the model on these two challenging scenes.

**Figure 9:**
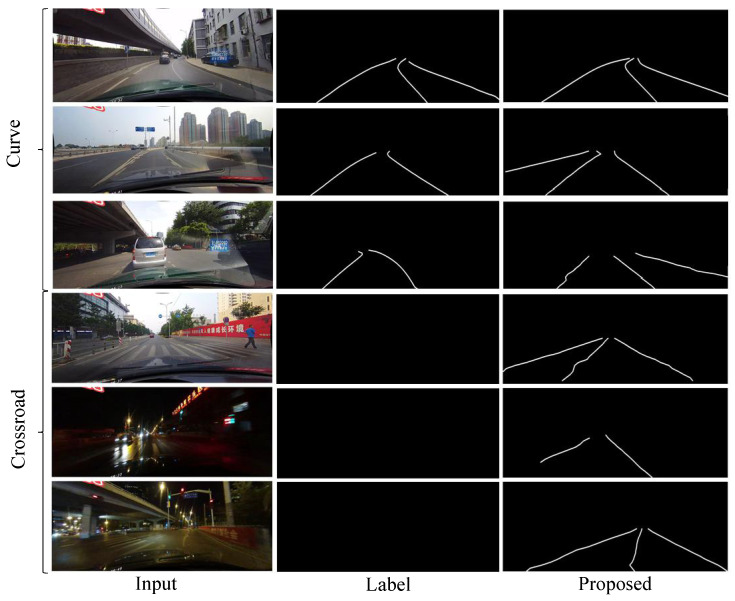
The lane detection results of the proposed model in the curved and crossroad scenes on CULane dataset. The first three rows are the results on partly curved scenes, and the last three rows show the results of our model in the crossroads. Note that the labels of the crossroad images are empty on the CULane.

As can be seen in Figure 9, we show some examples of curved lane lines and crossroads in the experimental results. The prediction result shown in the first row of the Figure 9 proves that our model has the ability to detect lanes in the curved lane lines scene. Meanwhile, we find some factors that negatively affect the indicators that are obtained by our proposed model for lane detection in the curved lane lines scene, by sorting out and analyzing the results of the experiment. The labels on the CULane dataset are manually labeled, and it is easy to appear that the lane lines in some images are not labeled. Like in the second row of Figure 9, our proposed model still can detect lane lines that are not marked in the label, but they are actually on the road. Furthermore, the proposed model sometimes detects more lane lines based on previous learning, which potentially exist in real scenes (the third row in Figure 9) but not labeled in CULane. The occurrence of these situations will reduce the F1-measure value to a certain degree.

In addition, lane detection at crossroad is difficult for all models, so FP is used to compare the lane detection results in this scene. In fact, as shown in Figure 9, there are no labels in the crossroad scenes in CULane dataset. The division of the drivable area of the crossroad is very vague. The lane ground truth is very hard to be annotated by human. However, our model can use the context information and vertical spatial information to detect more potential real lane lines, which will make it easier to mark places that are not lane lines as lane lines. It can result in a higher FP value for our model. Therefore, our model does not perform well in a crossroad driving scene. The FP of R-34-SAD is the lowest at the crossroad, but the performance of this model is also the lowest in the overall challenging scenarios. Perhaps the crossroad scenario has limited evaluation effect on model performance.

## 6. Conclusions and Future Works

In short, we propose a robust lane detection model inspired by the ERFNet for complex traffic driving scenes. In this work, we strengthen the use of contextual information and vertical spatial features in our model by two designed blocks. More contextual information and the useful vertical spatial features can significantly enhance unclear and occluded lane detection. After a number of verification experiments were analyzed, we conclude that the proposed model is more robust for unclear and occluded lane detection than others.

The limitation of these two blocks is that many of the information transmitted to the subsequent network is useless. We consider using the attention mechanism to select the region of interest, which can effectively filter information and accelerate the network’s inference time. The parameter size of the proposed model is 23.2 M, and the running time of processing one image with a single NVIDIA Titan XP 12 GB GPU (Santa Clara, California, USA) is 188.7 ms. This shows that although our model can robustly solve lane detection in the complex driving scenarios, but it still cannot meet real-time requirements. In future work, we will consider improving the model to perform lane detection in real time and make the model perform better in crossroad and curved scenes.

## Figures and Tables

**Table 1:** The specific structures of the modules used in this decoding branch.

Layer	Block Name	Channel
1	Downsampling block	16
2	Downsampling block	64
3	Down-convolution block	64
4	Merge block	128 → 64
5	Downsampling block	64 → 128
6	Vertical spatial convolution	64 → 128
7	dilated convolution block	128
8	Merge block	256 → 128

**Table 2:** The specific structures of the modules used in this decoding branch.

Layer	Block Name	Type	Channel
9	upsampling	transposed convolution	128 → 64
10	up-convolution block	Non-bottleneck-1D block	64
11	upsampling	transposed convolution	64 → 16
12	up-convolution block	Non-bottleneck-1D block	16
13	upsampling	transposed convolution	16 → 3

**Table 3:** The proportion of each scene on CULane dataset.

Scenario	Normal	Crowded	Night	No Line	Shadow	Arrow	Dazzle Light	Curve	Crossroad
Proportion	27.7%	23.4%	20.3%	11.7%	2.7%	2.6%	1.4%	1.2%	9.0%

**Table 4:** Results of the comparison of our model with similar network models. The bold scores are the best.

Network Model	ERFNet	SCNN	ERFNet + SCNN	Proposed
F1 (0.3)	80.4	**80.9**	79.8	80.6
F1 (0.5)	71.8	71.6	71.0	**71.9**

**Table 5:** Comparison of different dilation rate combinations. The bold scores are the best.

Dilation Rate	Dilation Rates [2, 4, 8, 16]	Dilation Rates [1, 1, 1, 1]	Dilation Rates [1, 2, 1, 4]
F1 (0.3)	80.5	80.4	**80.6**
F1 (0.5)	71.4	71.5	**71.9**

**Table 6:** The use of different forms of spatial convolution. The bold scores are the best.

Form	Spatial Convolution	Horizontalspatial Convolution	Vertical Spatial Convolution
F1 (0.3)	78.9	79.8	**80.6**
F1 (0.5)	70.5	68.7	**71.9**

**Table 7:** Comparison of the performances with different models on TuSimple. The bold scores are the best.

Module	FP	FN	Accuracy
ResNet-18	0.0948	0.0822	92.69%
ResNet-34	0.0918	0.0796	92.84%
ENet	0.0886	0.0734	93.02%
Proposed	0.1875	0.0467	**96.2%**

**Table 8:** Comparing the performances with different algorithms on CULane. We select an IoU threshold of 0.5 (F1 (0.5)) for this experiments. The FP is shown at the crossroads. Our experiment uses official evaluation metrics so that it can be compared fairly with other models. The bold scores are the best.

Category	SCNN	R-101-SAD	R-34-SAD	ENet-SAD	Proposed
Normal	90.6	90.7	89.9	90.1	**91.1**
Crowded	69.7	**70.0**	68.5	68.8	69.8
Night	66.1	**66.3**	64.6	66.0	66.2
No line	43.4	43.5	42.2	41.6	**44.6**
Shadow	66.9	67.0	67.7	65.9	**68.1**
Arrow	84.1	84.4	83.8	84.0	**86.4**
Dazzle light	58.5	59.9	59.9	60.2	**61.5**
Curve	64.4	65.7	**66.0**	65.7	63.9
Crossroad	1990	2052	**1960**	1998	2678
Total	71.6	71.8	70.7	70.8	**71.9**

## Data Availability

Not applicable.
